# Supplementary material for: Copy Number Variation of the SHE Gene in Sheep and Its Association with Economic Traits
Source: Animals (Basel). 2019 Aug 6;9(8):531. doi: 10.3390/ani9080531 (PMC6720781; doi:10.3390/ani9080531)
Supplement: Supplementary file 1 [file animals-09-00531-s001.pdf]

Table S1. Association of *SHE* gene CNV with growth traits in four sheep breeds.

Breed	Growth Traits	CNV Type (Mean ± SE)			*p*
		Loss	Normal	Gain	
CKS	body height (cm)	66.16 ± 0.97	66.84 ± 0.96	66.62 ± 0.96	0.491
	heart girth (cm)	90.00 ± 1.93	90.03 ± 1.92	89.22 ± 1.92	0.676
	body weight (kg)	54.33 ± 2.91	56.51 ± 2.88	54.61 ± 2.89	0.367
HS	body height (cm)	61.63 ± 0.30	62.32 ± 0.77	62.50 ± 0.75	0.452
	rump width (cm)	17.36 ± 0.09	17.50 ± 0.23	17.72 ± 0.23	0.354
	body slanting length (cm)	70.88 ± 0.31	70.83 ± 0.77	70.12 ± 0.77	0.658
	body weight (kg)	32.01 ± 0.37	32.92 ± 0.92	33.17 ± 0.92	0.388
STHS	body slanting length (cm)	58.78 ± 0.77	58.85 ± 0.87	59.24 ± 0.75	0.900
	body height (cm)	62.64 ± 0.53	63.04 ± 0.60	64.01 ± 0.52	0.164
	heart girth (cm)	71.16 ± 0.75	72.10 ± 0.85	72.81 ± 0.73	0.286
	Circumference of cannon bone (cm)	7.16 ± 0.09	6.98 ± 0.10	7.12 ± 0.08	0.433
	chest depth (cm)	27.61 ± 0.33	27.77 ± 0.37	27.48 ± 0.32	0.850
LTHS	body slanting length (cm)	76.51 ± 2.87	76.96 ± 1.71	77.00 ± 2.19	0.990
	chest width (cm)	21.69 ± 1.16	22.58 ± 0.69	22.99 ± 0.82	0.690
	body height (cm)	76.84 ± 2.64	78.33 ± 1.58	75.86 ± 2.02	0.573
	heart girth (cm)	90.23 ± 3.09	94.95 ± 1.71	98.56 ± 2.26	0.123
	circumference of cannon bone (cm)	8.01 ± 0.35	7.92 ± 0.21	7.44 ± 0.27	0.287
	chest depth (cm)	37.05 ± 2.14	35.03 ± 1.77	35.01 ± 1.60	0.697
	high at the cross (cm)	76.39 ± 7.02	82.80 ± 4.19	90.68 ± 5.37	0.258
	body weight (kg)	50.16 ± 5.94	55.83 ± 3.42	58.12 ± 4.22	0.590

CKS: loss (169), normal (86), gain (47). HS: loss (150), normal (24), gain (24). STHS: loss (65), normal (54), gain (70). LTHS: loss (6), normal (30), gain (25).

Table S2. The phenotypic variance explained by the CNV of *SHE* gene.

Breeds	Growth Traits	R2	Percentage
CKS	body length (cm)	0.0004	0.04%
HS	circumference of cannon bone (cm)	0.0240	2.40%
	heart girth (cm)	0.0390	3.90%
STHS	chest width (cm)	0.0004	0.04%
	high at the cross (cm)	0.0388	3.88%

CKS: loss (169), normal (86), gain (47). HS: loss (150), normal (24), gain (24). STHS: loss (65), normal (54), gain (70).

Table S3. The additive and dominance effect of the *SHE* gene CNV.

Breeds	Growth Traits (cm)	Type	Additive Effect Value	Dominant Effect Value
CKS	body length	Loss	−0.92	−0.31
		Normal	0.61	0.72
		Gain	2.14	−1.69
HS	circumference of cannon bone	Loss	−0.16	0.08
		Normal	0.28	−0.35
		Gain	0.72	1.58
	heart girth	Loss	−0.08	−0.01
		Normal	0.14	0.04
		Gain	0.36	−0.16
STHS	chest width	Loss	0.54	−0.62
		Normal	0.02	0.58
		Gain	−0.51	−0.55
	high at the cross	Loss	−1.14	0.60
		Normal	−0.55	−0.56
		Gain	1.07	0.52

CKS: loss (169), normal (86), gain (47). HS: loss (150), normal (24), gain (24). STHS: loss (65), normal (54), gain (70).
